# Industrial Clothing

**Published:** 1905-09-16

**Authors:** 


### The Hospital.

A Journal of

The Medical Sciences and Hospital Administration.

Vol. XXXVIII.—No. 990. SATURDAY, SEPTEMBER 16, 1905.

### Industrial Clothing.

It was a prevailing belief in this country, half a century ago, that the English, as compared with other civilised nations, were conspicuous by their cleanliness. At the time when the Great Exhibition of 1851 was being prepared, that most prolific of caricaturists, John Leech, had no more favourite subject for his pencil than the inevitable dismay and bewilderment of the expected foreign visitors in the presence of the apparatus for personal washing which they would everywhere encounter. Our household baths were to be regarded only as comfortable sleeping places; our larger breakfast cups were to be regularly utilised as toilet basins; and the milk jugs as ewers to accompany them. A cake of soap was to be mistaken for a sweetmeat, and was to occasion much dissatisfaction by the contrast between its appearance and its flavour. We heard of the Belgian servant, who had lived with an English family and disliked them. "They were such dirty people, they were always washing themselves." It is very curious to find, after the lapse of the few intervening years, how completely the aspect of things has changed. It is quite true that the fixed household bath is now looked upon as a necessity in families of a class which would scarcely even have thought of it half a century ago; but in the intervening time the personal cleanliness of Continental nations has been carried to a high point, while that of the English industrial classes, if it has not gone back, has at least remained stationary, and the consequent aggregations of filthy persons, intensified by a vast increase of population, have become serious impediments to the maintenance of a proper standard of public health. The resulting evils are being rendered more and more obvious by modern methods of locomotion. Fifty years, or even twenty years ago, the industrial classes walked to and from their work. To-day they ride in omnibuses and tram-cars; and very large numbers of cleanly and well-dressed people have no alternative but to do the same, and to submit to be shocked and offended by the dirty and evil-smelling garments of their fellow passengers; evil-smelling not only from contamination by the materials of labour, but also, and in a far greater degree, from prolonged contact with unwashed and perspiring human bodies. The County Council tram-cars contain copies of rules and regulations under one of which persons in offensively dirty clothing may, it is said, be excluded; but, unless it be in the case of chimney-sweeps in full uniform, there has probably never been an instance in which the rule has been enforced.

A party of Birmingham brassworkers has lately paid a visit of observation to Berlin, and a very interesting little book has been published, containing an account of what they saw. Their simple wonder at the personal cleanliness of persons of their own class in the Prussian capital, and at the cleanliness of their children and of their dwellings, is almost like the wonder of savages at the resources of civilisation. The same feelings would have been excited if they had gone to Paris or to Pittsburgh, to Vienna or to Chicago, and everywhere, out of England, they would not only have found the industrial classes cleaner in their persons than they are with us, but would have found also that the greater cleanliness of their persons was attended by greater cleanliness of their homes, and by a greater degree of self-respect than a dirty man can ever experience. The dirtiest of the travelling workmen in this country generally endeavour to make up for the absence of self-respect by an increase of self-assertiveness, and apparently consider it manly to be rude. The result is not only a scandal to civilisation, but it is also a manifest danger to public health. Wherever there are accumulations of dirt there must also be breeding grounds of disease; and nothing could be more conducive to the direct welfare of our industrial classes, and indirectly, at least, to the welfare of other classes also, than to raise them to the level of personal and domestic cleanliness which so many Continental nations have already attained. The first step towards reform might be taken by the County Council, in a steady enforcement and gradual tightening of their own rules about offensively dirty passengers, with a view to the ultimate establishment of that habit of keeping working clothes at the workshop, and of changing them before going home, which is almost universal in the United States and in many centres of Continental industry, and which might be introduced among ourselves with great advantage. Another step would be in the introduction of washing materials for working clothes, such as are commonly employed for the purpose in other countries. The English gentleman adopts his dress to his pursuit. The French artisan is content with his blouse, and has no desire to be "*Monsieur en paletot,*" but the English artisan is only happy when he is wearing a caricature of the garments of classes whose occupations are different from his own. Would it not be possible for "labour leaders" to bring a little common sense to bear upon this matter, and to tell a few home truths to the men whom they are supposed to influence? It is unfortunately the case that many "authorities" fall short of their duty in the enforcement of measures for the protection of the public health; but many important causes of disease must be looked for in the habits of the people. Dirty clothes, dirty habitations, and dirty bodies are the influences which, in this twentieth century, are at least among the chief causes of illness, of physical deterioration, and of premature mortality.

